# High clonal diversity of *Staphylococcus aureus* isolates from children’s playgrounds in Hungary

**DOI:** 10.1038/s41598-024-60481-0

**Published:** 2024-05-01

**Authors:** Andrea Horváth, Áron Tormássi, Szofia Hajósi-Kalcakosz, Annamária Huber, Judit Sahin-Tóth, Orsolya Dobay

**Affiliations:** 1 Institute of Medical Microbiology, Semmelweis University, Nagyvárad tér 4., 1089 Budapest, Hungary; 2 Department of Infectious Diseases, Heim Pál Children’s Hospital, Üllői út 86., 1089 Budapest, Hungary

**Keywords:** *Staphylococcus aureus*, Playground, Children, One health, MSSA-CC398, Bacteria, Infectious-disease epidemiology, Microbial ecology

## Abstract

*Staphylococcus aureus* is one of the most important human pathogenic bacteria and environmental surfaces play an important role in the spread of the bacterium. Presence of *S. aureus* on children’s playgrounds and on toys was described in international studies, however, little is known about the prevalence and characteristics of *S. aureus* at playgrounds in Europe. In this study, 355 samples were collected from playgrounds from 16 cities in Hungary. Antibiotic susceptibility of the isolates was tested for nine antibiotics. Presence of virulence factors was detected by PCR. Clonal diversity of the isolates was tested by PFGE and MLST. The overall prevalence of *S. aureus* was 2.81% (10/355) and no MRSA isolates were found. Presence of *spa* (10), *fnbA* (10), *fnbB* (5), *icaA* (8), *cna* (7), *sea* (2), *hla* (10), *hlb* (2) and *hlg* (6) virulence genes were detected. The isolates had diverse PFGE pulsotypes. With MLST, we have detected isolates belonging to ST8 (CC8), ST22 (CC22), ST944 and ST182 (CC182), ST398 (CC398), ST6609 (CC45), ST3029 and ST2816. We have identified a new sequence type, ST6609 of CC45. *S. aureus* isolates are present on Hungarian playgrounds, especially on plastic surfaces. The isolates were clonally diverse and showed resistance to commonly used antibiotics. These data reinforce the importance of the outdoor environment in the spread for *S. aureus* in the community.

## Introduction

*Staphylococcus aureus* is one of the most important human pathogens, causing a wide range of infections from localized suppurative infections as pneumonia, osteomyelitis, endocarditis to bacteremia and sepsis^1^.

Asymptomatic *S. aureus* colonization of healthy people is very frequent, especially in the nasopharynx: 15% of the population is continuously colonized with *S. aureus*, whereas intermittent colonization is described in 70% of individuals. In children, colonization rates are even higher, with peak incidence at 10 years of age^2^.

*Staphylococcus aureus* is transmitted from human to human or from animal to human directly with close physical contact and respiratory droplets; or indirectly via contaminated fomites. Inanimate objects serve as potential reservoir and source of infection both for community acquired and hospital acquired *S. aureus* infections^3^. *S. aureus* is remarkably resistant to environmental factors and may persist on surface of fomites for hours, days or even months. Length of the survival is influenced by several factors, including the material of the object, environmental conditions (temperature, humidity), amount of contaminating bacterium introduced to the surface and presence of bodily fluids^3^.

There are several studies evaluating the prevalence of *S. aureus* on indoor surfaces. Most of these reports focus on the presence of the pathogen in the hospital environment^4,5^. Outside of the healthcare system, numerous studies assessed the environmental presence of *S. aureus* in the households of known carriers of the bacterium and have found that the pathogen can persist on household fomites for up to three months^6,7^. Public spaces can also be sources of *S. aureus* transmission. Prevalence of *S. aureus* on non-hospital public indoor fomites (public transportation, public phones, ATM, door handles, surfaces in schools and universities) varies widely from 6.5% up to 91.4%^8^.

In spite of the ubiquitous nature and the importance of the bacterium, there is a limited number of studies describing the prevalence of *S. aureus* on public open-air surfaces, such as playgrounds and to our knowledge there is no data available on the prevalence of *S. aureus* on playgrounds from Europe.

This study aimed to evaluate the prevalence, antibiotic susceptibility, virulence pattern and clonal composition of *S. aureus* isolates from public outdoor playgrounds from Hungary.

## Material

### Environmental sampling

**Figure 1:**
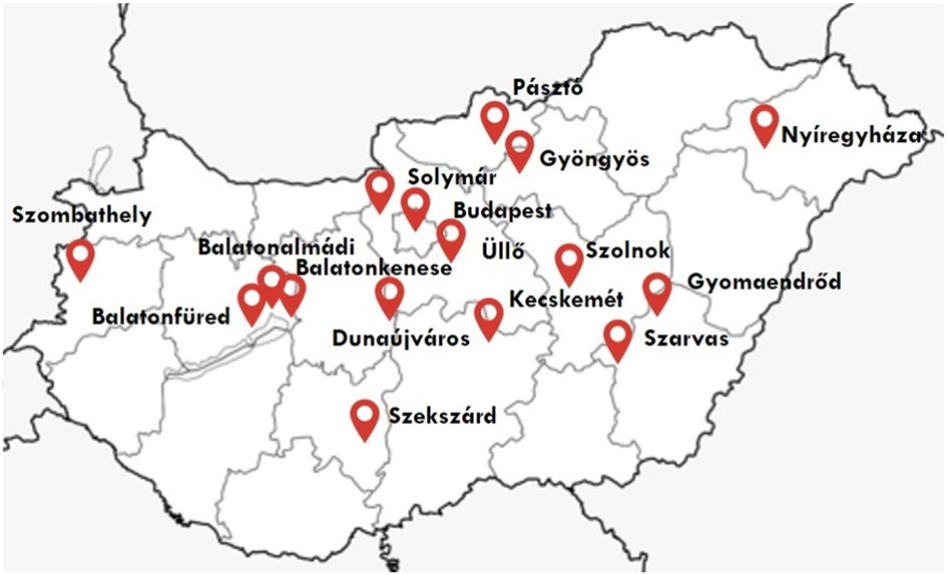
Location of sample collection (modified from^9^).

A total of 355 samples were collected from 24 public outdoor playgrounds in 16 cities of Hungary between September 2018 and September 2019 (Fig. 1). All samples have been collected in fair weather conditions (15–25 °C temperature, dry weather). The sampled surfaces were chosen as the most hand touched areas at the sites. Locations were chosen to represent frequently visited playgrounds from both small and large cities from different areas of the country. Surfaces were swabbed with sterile cotton swabs premoistened with sterile 0.9% sodium chloride solution. Samples were transferred to the laboratory in sterile, charcoal containing Amies transport medium (Transwab, Medical Wire & Equipment, Corsham, UK). Specimens were stored and transported at room temperature per the instructions of the manufacturer. Samples were plated within 24 h.

### Bacterial culture and identification

Samples were inoculated onto Columbia blood agar plates and onto Chromagar Staph aureus (CHROMagar, Paris, France). After an overnight incubation at 37 °C colonies showing typical species-specific morphology were chosen to produce pure cultures and were identified as *S. aureus* by catalase and latex agglutination test (Pastorex Staph-Plus Kit, Bio-Rad, Marnes-la-Coquette, France). Genotypic identification was based on the PCR detection of the species-specific thermonuclease gene (*nucA*), using primers described by Laub et al.^10^. The cycling parameters were as follows: 3 min at 94 °C, then 30 cycles of 94 °C 60 s, 54 °C 60 s, and 72 °C 30 s, and a final extension of 72 °C for 10 min. For all PCR gel electrophoresis, the GeneRuler 100 bp DNA ladder (ThermoFischer Scientific) was used. *S. aureus* ATCC 29213 was used as positive control. Confirmed *S. aureus* isolates were stored at − 80 °C on MAST CRYOBANK™ cryobeads (Mast Diagnostica, Bootle, UK).

### Antibiotic susceptibility testing

The MIC of the isolates was determined by the agar dilution method using an A400 multipoint inoculator (AQS Manufacturing Ltd., Southwater, UK) on Mueller–Hinton agar plates. Cefoxitin susceptibility was tested by disk diffusion method. The results were interpreted according the EUCAST guidelines^11^. The following antibiotics were tested: penicillin, cefoxitin, oxacillin, erythromycin, clindamycin, gentamicin, tetracycline, ciprofloxacin, levofloxacin and vancomycin. Inducible resistance to clindamycin was tested by ‘D test’ according to EUCAST guidelines^11^.

### Molecular characterization

PCR was carried out on all *S. aureus* isolates to detect the presence of the methicillin resistance gene *mecA*, and the following virulence factor genes: PVL genes (*lukS, lukF*), haemolysin A, B and G (*hla, hlb and hlg*) genes, Staphylococcus protein A (*spa*) gene, exfoliative toxin A and B (*eta, etb*) genes, toxic shock syndrome toxin (*tsst*), and Staphylococcus enterotoxin A, B, C (*sea, seb, sec*) genes as described previously^12^. For each virulence gene, the appropriate control strain was applied, obtained from the collection of the National Public Health Centre of Hungary. Bacterial DNA was isolated from the bacteria with the Quick-DNA Fungal/Bacterial Miniprep Kit (Zymo Research, California, USA). The DNA samples were stored at – 80 °C until further testing.

All *S. aureus* isolates were genotyped by pulsed-field gel electrophoresis (PFGE). The preparation of the genomic DNA for PFGE analysis included a 3-h lysis in lysostaphin and lysozyme, followed by an overnight lysis in proteinase K (Sigma Aldrich). The *Sma*I digestion was performed for 45 min (ThermoScientific FastDigest enzyme). For PFGE gel electrophoresis, we have applied the following running conditions: Pulse times: block 1, 5 s/15 s for 10 h, and block 2, 15 s/60 s for 11 h; at 14 °C. The CHEF DNA Size Standard 48.5–1000 kb Lambda Ladder (Bio-Rad) was used as a molecular size marker.

Multi-locus sequence typing (MLST) was performed on all isolates and Sequence Types (STs) were assigned using organism specific MLST database^13^. The PCR products for the MLST analysis were purified with the QIAquick PCR Purification Kit (Qiagen, Venlo, Netherlands). The final DNA concentration was measured by a NanoDrop Lite spectrophotometer (Thermo Scientific). PHYLOViZ software v2.0 was used to analyze relatedness of STs and to draw minimum spanning tree.

### Ethical approval

The Semmelweis University Regional and Institutional Committee of Science and Research Ethics has confirmed that no ethical approval is required for this study.

## Results

*Staphylococcus aureus* isolates were found in 10 samples (2.81%, 10/355), all positive samples were collected at playgrounds at Budapest (5.49%, 10/182). Seven out of the 10 samples were recovered from plastic surfaces, the remaining three from metal surfaces of playground equipment (swings, slides, monkey bars and seesaws).

### Virulence factors

**Figure 2:**
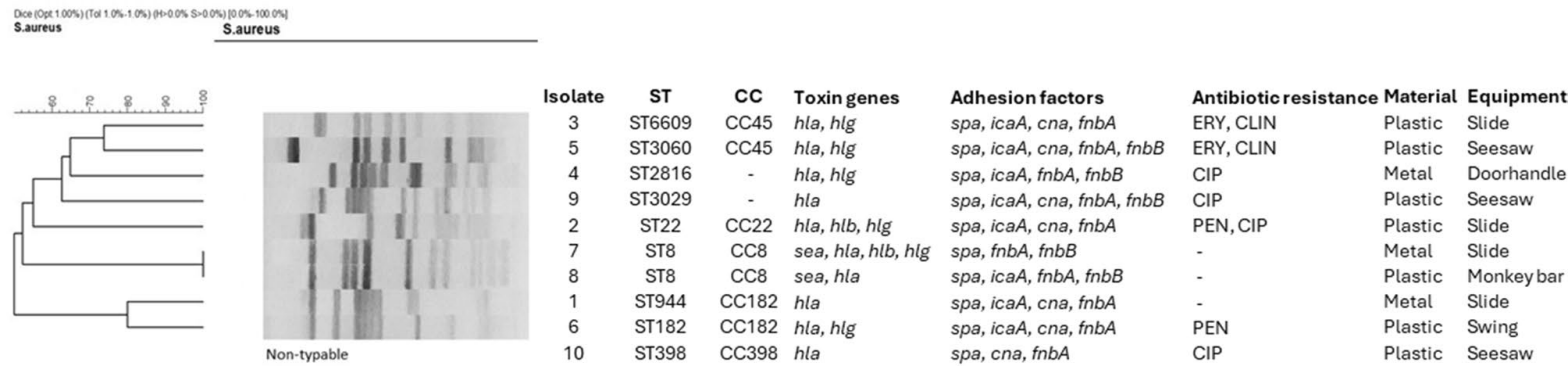
Clonal diversity, antibiotic resistance and virulence gene patterns of the isolates. PEN, penicillin; ERY, erythromycin; CLI, clindamycin; CIP, ciprofloxacin.

All ten isolates carried the haemolysin A gene (*hla*), whereas two were positive for haemolysin B (*hlb*) and six positive for haemolysin G (*hlg*). Two isolates possessed the staphylococcal enterotoxin A gene (*sea*). Genes encoding adhesion factors were present in all isolates, ten were positive for *spa* and *fnbA*, eight for *icaA*, seven for *cna*, and five for *fnbB*. None of the isolates was positive for *pvl* (Fig. 2).

### Antibiotic susceptibility

The isolates were susceptible to most of the tested antibiotics. Two isolates were resistant to penicillin, two to erythromycin and clindamycin and four isolates were resistant to ciprofloxacin. All isolates were susceptible to cefoxitin, oxacillin, gentamicin, tetracycline, levofloxacin and vancomycin (Fig. 2).

### Clonal diversity

**Figure 3:**
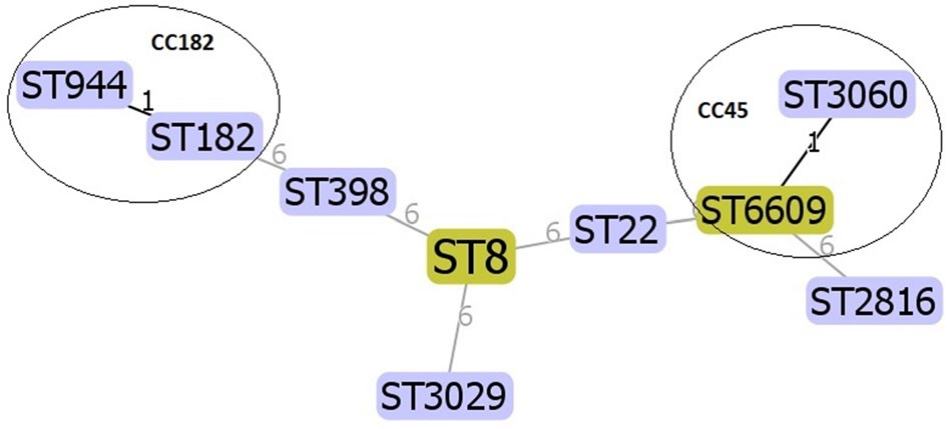
Genetic relatedness of the isolates with eBURST analysis based on MLST allelic differences.

Based on both MLST and PFGE results, our isolates proved to be clonally diverse (Figs. 2 and 3). According to MLST, two isolates belonged to sequence type (ST) 8 of clonal complex (CC) 8. One isolate each belonging to ST22 (CC22), ST398 (CC398), ST182 and ST944 (both in CC182), ST3060 (CC45), ST3029 and ST2816 were found. We have identified a new allelic combination, which was assigned a new sequence type: ST6609 (CC45) by the PubMLST Database, which is a single locus variant of ST3060. One isolate was non-typable by PFGE with *Sma*I digestion; this isolate belonged to ST398.

## Discussion

*Staphylococcus aureus* is the leading cause of invasive bacterial infections in children. Skin and soft tissue infections (SSTI), including impetigo, folliculitis, cellulitis, and cutaneous abscesses are the most frequent infections caused by *S. aureus* in the pediatric population, moreover, these localized infections can possibly disseminate to invasive, life-threatening infections^14^.

Children’s playground are generally high traffic areas where many visitors, adults and children, spend extended time. High-touch zones of playground equipment can easily become contaminated by microbes from humans and from the environment. According to a study evaluating the presence of bacteria and bodily fluids on various public surfaces, the highest contamination rates were found on surfaces from children's playground equipment and daycare centers. Children are especially prone to exposure to environmental contaminations due to frequent hand-to-mouth contact^15^. Children are exposed to many microbes during their stay at playgrounds and in parks. A study from Mexico found that 2.5% of children had *S. aureus* on their hand after one hour play at a playground, among other bacteria, viruses and protozoa^16^.

In a study from Ohio, USA, samples were collected from 10 outdoor playgrounds. *S. aureus* was found in 31.8% of the samples, 3.9% of the samples were positive for MRSA^17^. The most prevalent sequence type was ST5/USA100, a common clone in the USA, followed by ST8/USA300 isolates. Five livestock associated ST398 isolates were also found.

A study from Greece demonstrated the importance of the season (temperature, humidity) in the presence of pathogenic bacteria in soil of playgrounds, but not on equipment surface^18^. Chatziprodromidou et al. detected 5.1 CFU/g *S. aureus* in soil of playgrounds in cold wet season, however, have found no *S. aureus* during hot dry season^18^. Gastroenteritis outbreaks of children were linked to *Salmonella enterica* and *Salmonella typhimurium*, associated with playground sand in Austria and in Spain^19,20^. Another gastroenteritis causing bacterium, *Campylobacter jejuni* was isolated from 12.5% of the wild bird droppings collected at playgrounds in New Zealand. Authors have linked the frequent environmental presence of the bacterium at playground environment to high rate of campylobacteriosis in children under 5 years of age^21^. Presence of *Escherichia coli* is also frequent at playgrounds all over the world. In 2009, an outbreak of diarrhea-associated haemolytic uraemic syndrome, leading to the death of one child, was caused by a shiga toxin-producing *E. coli* strain in Germany. Likely, the place of the infection was the local playground^22^.

Whereas several studies have evaluated the presence of other—mostly Gram-negative—bacteria at playgrounds, to our knowledge this is the first study about *S. aureus* on playground equipment in Europe so far.

In this study, we have found 2.8% prevalence rate of *S. aureus* on outdoor playground equipment. This rate in much lower compared to a previous study from the USA^17^. Differences in climate, number of people visiting playgrounds and *S. aureus* carriage rates in the population may account for this variation.

All *S. aureus* isolates were recovered from playgrounds of the capital city, Budapest. Higher population density leading to higher numbers of playground visitors is likely to contribute to this result.

Most of our isolates (7/10) derived from plastic surfaces, whereas less *S. aureus* was found on metal surfaces (3/10) and no isolates were recovered from wooden equipment **(**Fig. 2). This is in accordance with a previous study testing the survival of MRSA on various surfaces. MRSA survived the longest on plastic and vinyl and for the least amount of time on wood^23^.

*Staphylococcus aureus* isolates in this study were generally susceptible to the tested antibiotics, however, some isolates had resistance to commonly used antibiotics, such as penicillin, erythromycin, clindamycin and ciprofloxacin (Fig. 2). Antibiotic resistance rates of isolates in this study were lower compared to clinical isolates, and similar to that found in a study describing *S. aureus* isolates asymptomatically colonizing Hungarian children^24^. The majority of our isolates (8/10) were susceptible to penicillin. Clinical isolates of *S. aureus* are generally resistant to penicillin; however, the proportion of penicillin susceptible *S. aureus* (PSSA) isolates has increased globally in recent years^25^. Macrolides are one of the most frequently used antibiotics in community-acquired infections of children; hence erythromycin and clindamycin resistance of environmental *S. aureus* isolates may lead to failure of empirical antibiotic therapy^26^.

All of our isolates carried the haemolysin A gene (*hla*), staphylococcal protein A gene (*spa*) and fibronectin binding protein A gene (*fnbA)* (Fig. 2). Several isolates have carried additional virulence factor genes, encoding for cytotoxins* (hlb, hlg)* and adhesion factors *(icaA, cna, fnbA*). Prevalence of virulence factor genes was lower compared to results of a study describing clinical isolates from *S. aureus* bloodstream infection in Hungary^12^. It is of particular interest that two isolates carried the *sea* gene encoding for staphylococcal enterotoxin A, potentially causing staphylococcal food poisoning.

It is well established that the population structure of *S. aureus* is clonal, the most dominant clones globally are clonal complexes (CC) CC5, CC8, CC22, CC30, and CC45^27,28^. Isolates of this study were diverse both by PFGE and MLST, they belonged to nine sequence types (ST) (Figs. 2 and 3). One isolate belonging to ST398 was non-typable with PFGE. This is a well-described characteristic of ST398 due to the methylation of the genome of this particular sequence type at the restriction site of *Sma*I enzyme, making ST398 resistant to standard PFGE typing^29^.

We have detected isolates of the most frequent *S. aureus* clones, including ST22, which is widespread all over Europe and has been increasingly reported from Asia and the Middle East in the recent years. ST22 is more prevalent in invasive infections compared to asymptomatic colonization^30^. Globally, isolates of CC45 are also very common, especially among nasal commensal isolates^31^. ST45 was described as the most frequent *S. aureus* strain from asymptomatic nasal colonization of Hungarian children^24^. In our study, we have found two CC45 isolates. One isolate belonged to ST3060, a single-locus variant of ST45. In the scientific literature, ST3060 *S. aureus* has been described on two occasions so far, both times from wild animals. A ST3060-*mecA*-MRSA isolate was recently identified from a hedgehog from Hungary. Hedgehogs are frequent carriers of *S. aureus* and they are prevalent in both rural and populated areas, have access to human food waste and playgrounds as well, and they may serve an important role in transmission of bacteria between wildlife, environment and humans. ST3060 *S. aureus* was also described from white storks exposed to human waste in Spain^32^. Migratory birds, such as storks play an important role in international and intercontinental dissemination of human pathogenic bacteria^33^ as their feces contaminates environment of humans, including playgrounds^21^. Various Staphylococci were described in feces of wild birds, suggesting their role in the carriage and dissemination of resistant bacteria into the environment^34^. Furthermore, in this study, we have identified a new sequence type, ST6609, a single locus variant of ST3060, belonging to CC45.

Two isolates of this study belonged to ST8 that is among the most prevalent clones in North America and from there has spread to all over the world^35^.

Two of our isolates belonged to CC182 (ST182 and ST944). ST182 has worldwide distribution and has been identified from asymptomatic colonization of adults and children, as well as invasive infections and animals^36^. ST944 is a single-locus variant of ST182 that has been described from asymptomatic carrier adults and schoolchildren and clinical isolates in China and in Portugal^37,38^.

Moreover, we have identified one isolate belonging to ST398 of CC398. Methicillin resistant strains of ST398 are important livestock associated pathogens of worldwide distribution, whereas methicillin susceptible ST398 isolates are adapted to humans, are primarily transmitted from human to human, with the majority of the infections reported from Europe and China^39^. However, wild animals, including the migratory white storks have also been described to carry MSSA-CC398^40^.

We have identified two singleton *S. aureus* isolates (ST2816 and ST3029), that differ from all other existing STs by at least two alleles, and do not belong to any existing CCs. ST2816 has been described from clinical isolates and asymptomatic colonization in Kuwait and in Saudi Arabia^41,42^. ST3029 has only been isolated in Malaysia, from skin infection so far^43^.

A limitation of our study is that all samples were collected within Hungary, therefore no global European picture can be extracted. Further studies are needed to get a better view on the prevalence and characteristics of *S. aureus* isolates on European playground and their potential role in colonization and infections of children.

## Conclusions

The presence of *S. aureus* isolates on playground equipment highlights the role of public outdoor playgrounds in the transmission of *S. aureus* in the community. Antibiotic resistance and the many virulence factors of the identified *S. aureus* isolates underline their potential as pathogens of pediatric infections. In line with the One Health concept, not only humans, but also wild animals, such as migratory birds may introduce human pathogenic bacteria onto playground surfaces, and these environmental reservoirs can serve as contact points for exchange of pathogenic bacteria between humans, animals and the ecosystem. The *S. aureus* isolates in this study showed high clonal diversity: both representatives of important international *S. aureus* clones and minor, rarely identified STs were present simultaneously with no dominant clone identified, emphasizing the importance of public open-air playgrounds introduction of different bacterial clones into the community.

## Data Availability

All data generated or analysed during this study are included in this article.
